# The Efficacy of Bacteriocins Against Biofilm-Producing Bacteria Causing Bovine Clinical Mastitis in Dairy Farms: A New Strategy

**DOI:** 10.1007/s00284-023-03324-x

**Published:** 2023-05-31

**Authors:** Ismail Raheel, Asmaa N. Mohammed, Asmaa Abdrabo Mohamed

**Affiliations:** 1 Department of Bacteriology, Mycology and Immunology, Faculty of Veterinary Medicine, Beni-Suef University, Beni-Suef, 62511 Egypt; 2 Department of Hygiene, Zoonoses and Epidemiology, Faculty of Veterinary Medicine, Beni-Suef University, Beni-Suef, 62511 Egypt; 3 Veterinarian at the Directorate of Veterinary Medicine, El-Fayoum Governorate, Egypt

## Abstract

Using an alternative bio-product is one of the most promising ways to control bovine mastitis and avoid new intra-mammary infections. The aims of this study were to ascertain the prevalence of biofilm-forming bacteria responsible for causing clinical mastitis in dairy herds and to assess the effectiveness of bacteriocins, produced by *Bacillus subtilis*, in controlling the growth of these bacteria in the milk of animals. A total of 150 milk samples were collected from cows and buffalos suffering from mastitis and the etiological agents were isolated and identified by the VITEK-2-COMPACT-SYSTEM®. Additionally, the capability of the bacterial isolates to produce biofilms was determined. RT-PCR was used to detect enterotoxin-producing genes (*sed* and *seb*), resistance genes (*mecA* and *blaZ*), and biofilm-associated genes (*icaA* and *fnbA*) in the isolated bacteria. The susceptibility patterns of the bacterial isolates to bacteriocins were assessed using an agar well-diffusion assay. *S. aureus* was significantly more capable of producing biofilms than coagulase-negative *Staphylococcus* isolates. *S. ubris* was the strongest biofilm producer among the *Streptococcus* species. The sensitivity profiles of the *Staphylococcus* spp. (*S. aureus* and coagulase-negative *Staphylococcus*) and their biofilm producers to bacteriocins were significantly higher (100% and 90%, respectively) at the same concentration. Bacteriocins had a lethal effect on *Staphylococci*, *Streptococci*, and biofilm development at a dose of 250 µg/mL. In dairy farms, bacteriocins are a viable alternative treatment for the prevention and control of bovine clinical mastitis.

**Supplementary Information:**

The online version contains supplementary material available at 10.1007/s00284-023-03324-x.

## Introduction

Bovine mastitis is the most prevalent disease affecting dairy cattle, causing financial losses and detrimentally affecting animal welfare, production, food safety, and the quality of milk [1, 2]. It can be caused by various Gram-positive and Gram-negative bacteria, such as *Staphylococcus aureus*, *Streptococcus agalactiae*, and *Mycoplasma* species. They can be infectious or environmental, for example, *Enterococcus* spp., *Escherichia coli,* coagulase-negative* Staphylococcus* (CNS), and *Streptococcus uberis*. To manage new cases of mastitis, general sanitation practices, such as disinfecting the teats post-milking, improving milking hygiene, and maintaining milking equipment, must be employed. Thus, natural remedies, especially alternative medicines, are particularly important for the prevention and treatment of bovine mastitis [3].

*S. aureus* is the most common Gram-positive bacteria associated with different types of clinical and subclinical mastitis [4]. It is primarily found in persistently infected mammary glands; therefore, keeping the udder clean during milking can help shield healthy cows from diseased cows, lowering the infection rate [5]. *S. agalactiae*, found in the environment of dairy cows and the gastrointestinal tract of cattle, causes infectious mastitis [6]. It can be transferred via milking machines and through the fecal–oral route, notably through contaminated drinking water. In fact, the mammary gland may become infected by germs found in the cow’s bedding area and on the milking apparatus [7].

Biofilms are organized bacterial populations attached to biotic or abiotic surfaces that constitute a self-produced matrix, which includes exopolysaccharides, proteins, teichoic acids, enzymes, and extracellular DNA [8]. Before adhesion is facilitated by cell wall-associated structures (flagella, fimbriae, and pili), biofilm formation begins with bacterial attachment to an abiotic surface via hydrophobic or electrostatic interactions. In conjunction with this adherence, polymer bridges between bacteria and the surface are frequently formed [9, 10]. Teat dips used nowadays in commercial pre- and post-milking processes include chlorine, hydrogen peroxide, and iodine. Despite being efficient, these substances could seriously irritate the skin [11]. Therefore, it is necessary to seek about more natural substitutes that can be utilized in combination or in addition to the current chemical materials.

Gram-positive rod- or cocci-shaped facultative anaerobes called lactic acid bacteria are being increasingly investigated for their ability to create inhibitory compounds resembling bacteriocins, small antimicrobial peptides that are active against various bacteria [12, 13]. Several bacteriocins have been characterized in terms of their structure, mode of action, and range of inhibitory activity [14]. Bacteriocins of lactic acid bacteria are classified as extracellularly produced primary or modified products of bacterial ribosomal synthesis, which have a bactericidal activity [15]. The action of bacteriocins based on disrupting membranes of bacteria and it has a net positive charge that, despite their diversity as peptides, allows them to fold into an amphiphilic shape when they come into contact with bacterial membranes [16]. The production of bacteriocins is normally performed in complex growth media: De Man, Rogosa, and Sharpe (MRS) broth [17]. This study was aimed at controlling the bacterial populations that cause clinical mastitis in dairy animals. We determined the prevalence of biofilm-producing mastitis-causing bacteria in dairy herds, identified biofilm-associated genes, and assessed the efficacy of bacteriocins produced by *Bacillus subtilis*, as natural alternatives to antimicrobial agents, against all isolated bacterial strains.

## Materials and Methods

### Study Site and Period

The study was conducted on both lactating cows and buffalos at private dairy farms in El-Faiyum Governorate between May 2019 and March 2021. Lactating animals were housed in an earthen-floored cow house system. Following the recommendations of the “National Mastitis Council,” the udder of each lactating animal was examined for the presence of clinical signs of mastitis, such as asymmetry, hotness, swelling, or any physical changes prior to sampling. This was followed by palpation to look for injury, atrophy, fibrosis, or inflammatory swelling. The dairy farms under investigation had minimal to moderate hygiene measures.

### Study Design

The study’s protocol was designed to estimate the prevalence of clinical mastitis in various lactating animals. In addition to examining their capacity to create biofilms, the most prevalent Gram-positive cocci that caused clinical mastitis in dairy farms were isolated and identified. Next, we assessed the effectiveness of bacteriocins against mastitis-causing, biofilm-forming bacteria to examine if they could be used as natural therapeutics to treat bovine clinical mastitis.

### Collection of Milk Samples

Milk samples were obtained aseptically as described earlier [18]. The udder and tips of the teat orifice were thoroughly cleaned with water and soap and dried with a sterilized cloth. The teats were cleaned with 70% alcohol. The first few streams of milk were excluded and milk samples (*n* = 150) were collected in sterile, screw-capped McCartney bottles, labeled, serialized, and transported immediately to the lab on ice for microbial analysis.

### Isolation and Identification of Clinical Mastitis-Causing Pathogenic Bacteria

After being incubated at 37 ℃ for 18–24 h, milk samples were centrifuged at 3000 rpm for 15 minutes. The fluid supernatant and the cream layer were discarded. A small amount of the sediment was extracted and cultured in tryptone soya broth for 18–24 h at 37 ℃. Loopfuls of broth were cultured on mannitol salt agar, Baird–Parker agar (to examine *Staphylococcus* spp.) and modified Edwards medium (to examine *Streptococcus* spp.) for 24–48 h at 37 ℃. For identification, bacteriological films were prepared, stained by Gram’s stain, and studied under a microscope. While suspected *Streptococcus* isolates were identified as Gram-positive cocci that were arranged either singly or in chains, suspected *Staphylococcus* isolates were identified as Gram-positive cocci occurring as singles, pairs, or mostly as irregular clusters (like bunches of grapes). Pure colonies that had been confirmed were transferred to tryptone soya agar and cultured for 24–48 h at 37 °C. Before biochemical identification of the isolates, their colony morphology and purity were assessed a second time according to Quinn et al. [18]. Additionally, VITEK-2-COMPACT-SYSTEM® (BioMérieux) was used to confirm the identity of the bacterial isolates. RT-PCR was also performed on different *Staphylococcus* and *Streptococcus* isolates to determine four genes: two enterotoxin-producing genes (*sed* and *seb*) and two resistance genes (*mecA* and *blaZ*). The primer sequences and sizes of PCR amplicons are illustrated in (S1) [19–21].

### Detection of Biofilm-Forming Bacteria on Yeast Extract–Casamino Acid Agar Supplemented with Congo Red (YESCA CR)

Pure colonies of the bacterial isolates were streaked on Luria–Bertani agar and incubated for 48 h at 37 ℃. A single colony was selected using a sterilized bacteriological loop, streaked onto YESCA CR agar, and cultured for 48–72 h at 25 ℃. The development of biofilms was investigated according to Zhou et al [22]**.** Pink or white color of the bacterial colonies indicated a failure to uptake the stain (negative for biofilm formation), while a red color indicated successful uptake of the dye (positive for biofilm formation).

### Detection of Biofilm-Associated Genes

RT-PCR was conducted on many distinct *Streptococcus* and *Staphylococcus* isolates to identify two biofilm-related genes: *icaA*, which encodes an *N-*acetylglucosaminyltransferase, and *fnbA*, which encodes fibronectin-binding protein A. The primer sequences used and amplicon sizes are summarized in S1 [23, 24].

The published sequence of the *icaA* and *fnbA* locus in GenBank was used to designate the primers for the *icaA* and *fnbA* genes. For *icaA* amplification, AF (5′-CCT AAC TAA CGA AAG GTA G-3') and AR (5′-AAG ATA TAG CGA TAA GTG C-3′) and for *fnbA* amplification, DF (5′-CAT AAA TTG GGA GCA TCA -3′) and DR (5′-ATC AGC AGC TGA ATT CCC ATT -3′) primers were used. The *icaA* and *fnbA* genes were used in the PCR to produce some products of 1315 bp and 127 bp, respectively. Ten μl of the rapidly extracted DNA were used as a template in a 50-μl PCR mixture containing 1X PCR buffer (50-mm KCl, 20-mM Tris–HCl), 5 μl of 25-mM MgCl2, 5 μl of 10-mM deoxynucleoside triphosphate (dNTP) mix, 1 μl of 20-μM each primers, and 1U of Taq DNA polymerase. The buffers and enzymes used in the assay were obtained from Fermentas Inc. The amplification of DNA was performed as follows: 92 ℃ for 5 min of initial denaturation; 30 cycles of 92 ℃ for 1 min, 49 ℃ for 1 min. and 72 ℃ for 1 min; and a final extension at 72 ℃ for 7 min. Amplicons were loaded onto 1.5% Agarose Gel containing 1-μg/ml ethidium bromide. The presence and molecular weight of the amplified DNA fragments were confirmed by agarose gel electrophoresis and visualized under UV light.

### Synthesis and Purification of Bacteriocins Produced by Lactic Acid-Fermenting Bacteria

*B. subtilis* stock was prepared by inoculating 10 mL of De Man, Rogosa, and Sharpe (MRS) broth with 0.1 mL of fresh lactic acid bacteria cultures and incubating for 12 h at 37 ℃. Next, 1 mL of this pre-culture was inoculated into 100 mL of MRS broth and incubated for 24 h at 37 ℃ [25]. The isolates grown on MRS broth for 48 h at 37 ℃ were centrifuged at 8000 rpm for 30 min at 4 ℃ to extract bacteriocins. The crude extract, labeled as the cell-free supernatant, was purified by filter sterilization through a 0.22-µm filter (Merck Millipore Ltd., Cork, Ireland) [26, 27]. The critical dilution method in 10-mM phosphate-buffered saline at pH 6.5 was used to recover the bacteriocins. The cell-free supernatant was adjusted to pH six with 1-M NaOH and heated at 80 ℃ for l0 min to deactivate extracellular proteases and hydrogen peroxide.

### Determining the Antibacterial Efficacy of Bacteriocins Using Agar Well-Diffusion Assay

The sensitivity profiles of biofilm-producing strains (*n* = 43) and pathogenic bacterial isolates (*n* = 49), obtained from animals suffering from mastitis, were examined in the presence of bacteriocins using an agar well-diffusion assay. All bacterial strains were freshly isolated and inoculated into brain heart infusion medium comprising 1.5% agar (w/v) at 1 × 10^5^ CFU/mL using a pour-plate method. Bacteriocin extract (25 µL), prepared as previously described, was poured into wells in the agar that had been perforated to a diameter of 5 mm. Each well carried a different concentration of bacteriocins: 50, 100, 150, or 250 g/mL. The plates were incubated for 24 h at 37 ℃ after the bacteriocins had been allowed to diffuse overnight at 4 ℃. According to Godoy-Santos et al. [28], all plates were inspected for the presence of zones of clearing, identified from the greatest dilution displaying an inhibition zone with a diameter ≥ 9 mm. The procedure was carried out in triplicates.

### Microscopic Analysis of Biofilm and Anti-Biofilm Activity of the Pathogenic Strains Using FESEM

On a sterilized acrylic strip, biofilm-producing bacteria were cultivated overnight. Thereafter, the samples were rinsed in 0.1-M buffer sodium cacodylate (Merck KGaA, Darmstadt, Germany) and then dehydrated through serial transfers in ethyl alcohol solutions of various concentrations for 30 min each. The specimens were mounted on metal stubs, after being left at room temperature for 24 h and a sputter coating equipment covered them with a layer of gold, while they were under vacuum (JEOL, JPC 1600, JEOL companies, Japan). At the National Research Center (Cairo, Egypt), the specimens were seen using field emission scanning electron microscope (FESEM; JEOL, JEOL Ltd., Japan) after being coated with gold.

### Data Analysis

All data were assembled for statistical analyses using the Statistical Package for the Social Sciences software. A non-parametric test (chi-squared test) was used to evaluate the antibacterial effectiveness of *B. subtilis* bacteriocins against all bacterial isolates. Meanwhile, one-way ANOVA test was used to determine the diameter of inhibition zone (mm) of testing bacteriocins against gram-positive cocci isolates. *P* ≤ 0.05 was considered statistically significant.

## Results

### Prevalence and Distribution Rate of Clinical Mastitis-Causing Gram-Positive Cocci Among the Dairy Farms

**Fig. 1:**
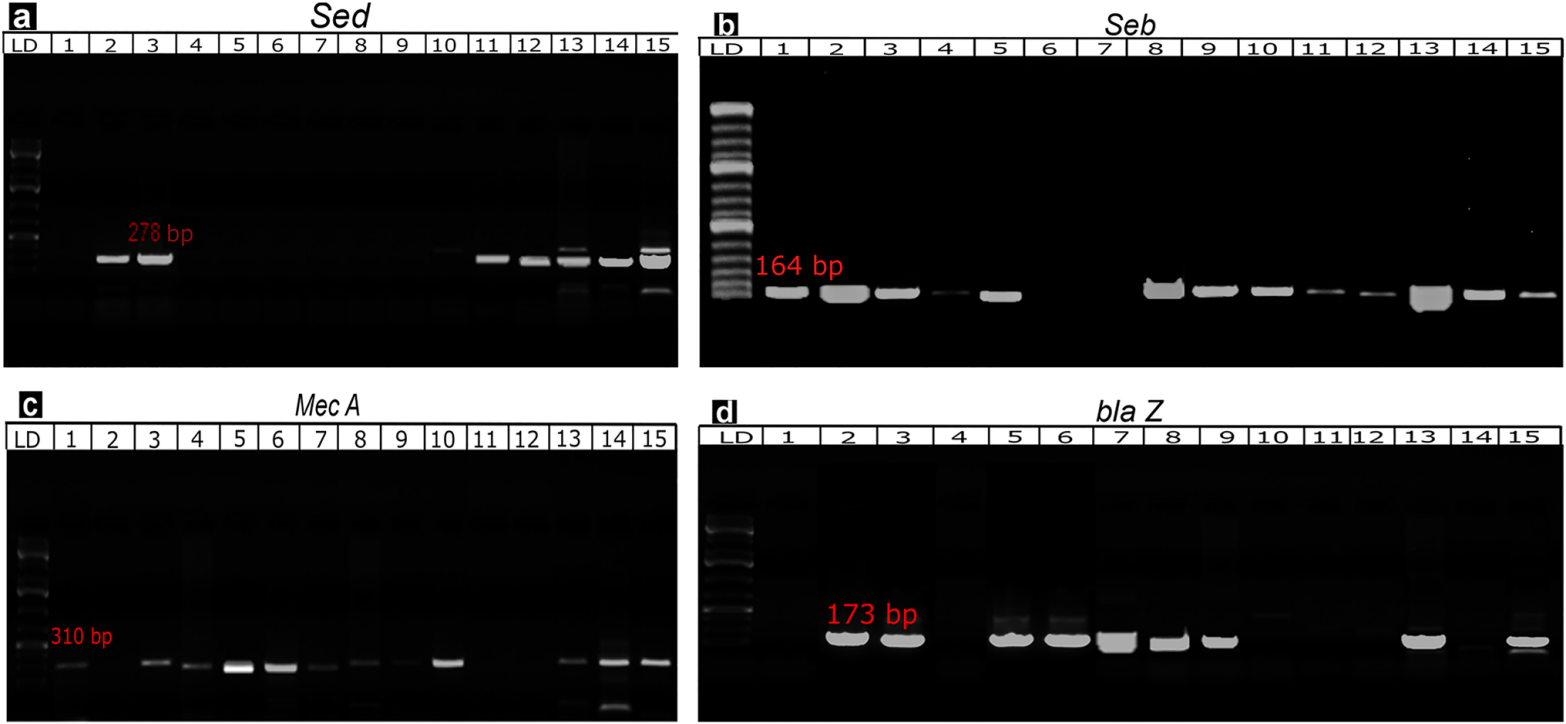
The *sed* gene (**a**) was amplified by PCR to obtain a 278-bp amplicon. Lanes (2, 3) show a positive result, LD stands for molecular size ladder. For the *Seb* gene (**b**), a 164-bp amplicon was expected. Lanes (1–3, 5, 8–10) show a positive result. For the *mecA* gene (**c**), a 310-bp amplicon was expected. Lanes (1, 3–10) show a positive result. For the *blaZ* gene **(d)**, a 173-bp amplicon was expected. Lanes (2, 3, 5–9) show a positive result

The prevalence of clinical mastitis-causing gram-positive cocci on dairy farms is shown in Table 1. Staphylococcus spp. were significantly more prevalent in the milk of buffalo with mastitis (29/36, 80.55%) than in that of cows with mastitis (52/78, 66.67%) at *P* ≤ 0.05; the opposite trend was observed for Streptococcus spp. (7/36; 19.44% vs26/78, 33.33%, respectively). Furthermore, enterotoxin- and resistance-related genes were detected by RT-PCR (Fig. 1). The *sed, seb*, *mecA, and blaZ* genes were amplified to give 278-bp-, 164-bp-, 310-bp-, and 173-bp-long amplicons, respectively (Fig. 1a–d).

**Table 1:** The prevalence rate of clinical mastitis-causing gram-positive cocci in dairy farms

Milk samples examined	Total examined no	Total positive no. (%)	Prevalence of bacterial isolates (No. %)			
			*Staphylococcus* spp.		*Streptococcus* spp.	
			Positive no	%	Positive no	%
Cattle	100	78 (78.0)	52	66.67	26	33.33
Buffalo	50	36 (72.0)	29	80.55	7.0	19.44
Total	150	114 (76.0)	81	71.05	33	28.95

The chi-square association of prevalence of Gram-positive cocci isolates among mastitis animals is statistically significant at *χ*^2^ = 126, *P* ≤ 0.05

The distribution of *Staphylococcus* and *Streptococcus* spp. isolates among the animals investigated is displayed in Table 2. The most common *Staphylococcus* spp. isolate found in cow and buffalo milk was CNS (63.46% and 62.07%, respectively) followed by *S. aureus* (36.54% and 37.93%, respectively). For *Streptococcus* spp., *S. agalactiae* was most prevalent in cow milk (34.61%) followed by *S. dysgalactiae* and *E. faecalis* (19.23% each), while *S. dysgalactiae* and *E. faecalis* were most abundant in buffalo milk (28.57% each) followed by *S. agalactiae, S. ubris,* and *S. lactarius.*

**Table 2:** Frequent distribution of gram-positive cocci isolates from clinical mastitic animals

Milk samples examined	*Staphylococcus* spp. No. (%)		*Streptococcus* spp. No. (%)				
	*S. aureus*	*CNS*	*S. agalactiae*	*S. dysgalactiae*	*S. ubris*	*E. faecalis*	*S. lactarius*
Cattle	19 (36.54)	33 (63.46)	9 (34.61)	5 (19.23)	4 (15.38)	5 (19.23)	3 (11.54)
Buffaloes	11 (37.93)	18 (62.07)	1 (14.28)	2 (28.57)	1 (14.28)	2 (28.57)	1 (14.28)
Total	30 (37.04)	51 (62.96)	10 (30.30)	7 (21.21)	5 (15.15)	7 (21.21)	4 (12.12)

The frequent distribution of Gram-positive cocci isolates among mastitis animals is statistically significant at *χ*^2^ = 96, *P* ≤ 0.05

### Detection of Biofilm-Related Genes of Gram-Positive Cocci Using RT-PCR

**Fig. 2:**
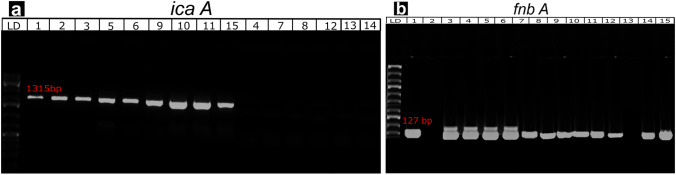
The *icaA* gene was amplified by PCR and a 131-bp amplicon was expected (**a**). Lanes (8, 9) show a positive result. For the *fnbA* gene, a 127-bp amplicon was expected (**b**). Lanes (11, 12, 14, 15) show a positive result. LD stands for molecular size ladder

**Fig. 3:**
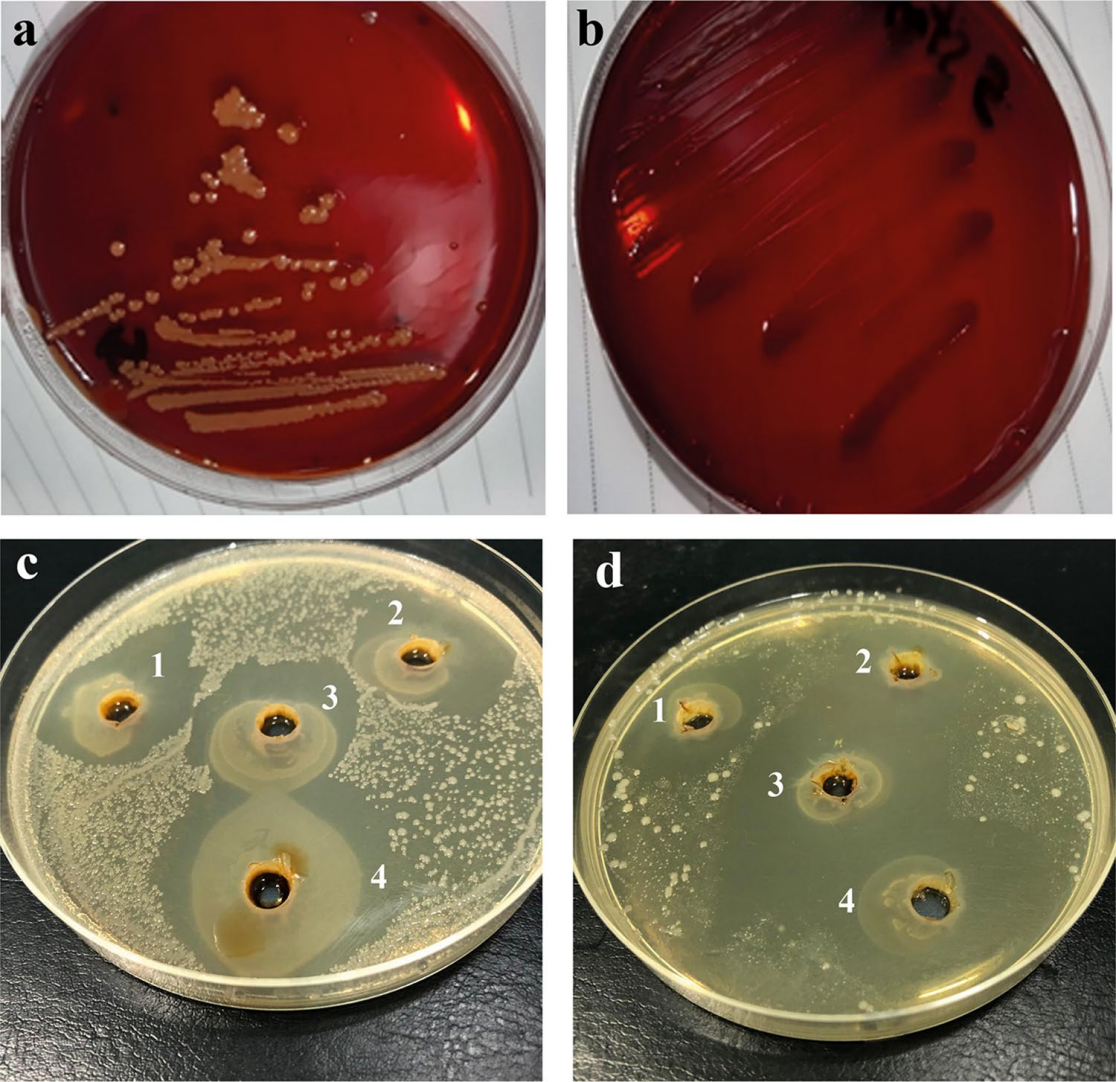
Biofilm-producing *Staphylococcus* spp. (**a**) and *Streptococcus* spp. (**b**) on YESCA CR agar. The red-colored colonies tested positive for biofilm formation. The procedure was carried out in triplicates**.** In addition, the sensitivity profile of the biofilm-producing strains of *Staphylococcus* and *Streptococcus* spp. to bacteriocins at different concentrations (1, 2, 3, and 4; 50, 100, 150, and 250 µg/mL, respectively) (Fig. 3c–d)**.** The diameter of the inhibition zone was determined using one-way ANOVA test. The inhibition zone of tested bacteriocins at 250 µg/mL against *Staphylococcus* spp. was significant 27.6 ± 0.21 mm (**c**) and 30.4 ± 0.15 mm for *Streptococcus* spp. (**d**)

The ability of *S. aureus* to form biofilms was significantly higher than that of CNS isolates (86.67% and 74.51%, respectively) (Table 3). *S. ubris* was the most potent *Streptococcus* species producing 100% of the biofilms, followed by *E. faecalis, S. agalactiae*, and *S. dysgalactiae* (71.43%, 70.0%, and 57.14%, respectively). Additionally, biofilm-related genes were found by RT-PCR (Fig. 2a–b): the *icaA* and *fnbA* genes were amplified to give 131-bp- and 127-bp-long amplicons. Moreover, the red coloration of the bacterial colonies on YESCA CR agar confirmed the presence of *Staphylococcus* and *Streptococcus* spp. (Fig. 3a–b) that form biofilms on the agar. The antimicrobial activity of bacteriocins against *Staphylococcus* spp. is shown in Table 4. The sensitivity of *S. aureus* to bacteriocins was significantly high (100%) at a concentration of 250 µg/mL compared with the other tested concentrations at *P *≤ 0.01, while that of CNS was 90% at the same concentration. At 150 µg/mL, their sensitivity to bacteriocins did not exceed 80%. In the case of *Streptococcus* spp., bacteriocins had a 100% lethal effect against *S. ubris* at 250 µg/mL, followed by *S. agalactiae, S. dysgalactiae,* and *E. faecalis* (90%, 80%, and 80%, respectively). Comparatively, the sensitivity of *S. lactarius* to bacteriocins did not exceed 75% at the same concentration

**Table 3:** The percentage of biofilm-producing gram-positive cocci among all *Staphylococcus* spp. and *Streptococcus* spp. isolates

*Bacterial isolates*	Total examined no	The percentage of biofilm-producing bacteria	
*Staphylococcus* spp.		No	%
*S. aureus*	30	26	86.67
*CNS*	51	38	74.51
Total	81	64	79.01
*Streptococcus* spp*.*			
*S. agalactiae*	10	7	70.0
*S. dysgalactiae*	7	4	57.14
*S. ubris*	5	5	100
*E. faecalis*	7	5	71.43
*S. lactarius*	4	2	50
Total	33	23	69.7

The percentage of biofilm-producing Gram-positive cocci isolates among mastitis animals is statistically significant at *χ*^2^ = 164, *P* ≤ 0.05

**Table 4:** The antimicrobial efficacy of bacteriocins against clinical mastitis-causing gram-positive cocci

Bacteriocins concentration (µg/mL)	*Staphylococcus* spp.				*Streptococcus* spp*.*										*P* value
	*S. aureus*		*CNS*		*S. agalactiae*		*S. dysgalactiae*		*S. ubris*		*E. faecalis*		*S. lactarius*		
	S	R	S	R	S	R	S	R	S	R	S	R	S	R	
50	50	50	40	60	40	60	50	50	30	70	60	40	50	50	0.05
100	70	30	60	40	50	50	60	40	40	60	60	40	50	50	0.04
150	80	20	80	20	70	30	80	20	60	40	80	20	75	25	0.02
250	100	0.0	90	10	90	10	80	20	100	0.0	80	20	75	25	0.01

*S* Susceptible, *R* Resistant; *S. aureus* (*n* = 10)*; CNS* (*n* = 10); *S. agalactiae* (*n* = 10); *S. dysgalactiae* (*n* = 5); *S. ubris* (*n* = 5); *E. faecalis* (*n* = 5); *S. lactarius* (*n* = 4)

Susceptible: means absence of microbial growth on agar plates. Resistant: means survival of microbial growth on agar plates

### Evaluating the Efficacy of Bacteriocins Against Biofilm-Producing Bacteria Using Agar Well-Diffusion Assay

The sensitivity pattern of biofilm-producing *Staphylococcus* spp. to different doses of bacteriocins is shown in Table 5. Biofilm-producing *S. aureus* and CNS were highly sensitive (90%) to bacteriocins at 250 µg/mL compared with the other tested concentrations while their sensitivity did not exceed 80% toward 150-µg/mL bacteriocins. On the other hand, 250 µg/mL bacteriocins produced a 100% lethal effect against biofilm-producing *S. ubris* followed by *S. agalactiae, E. faecalis,* and* S. dysgalactiae* (85.71%, 80%, and 75%, respectively). The sensitivity of biofilm-producing *S. lactarius *did not exceed 75% at the same bacteriocin concentration. The sensitivity profile of the biofilm-producing strains of *Staphylococcus* and *Streptococcus* spp. to different concentrations of bacteriocins is displayed in Fig. 3c–d. The diameter of the inhibition zone at 250 µg/mL of bacteriocins was 27.6 ± 0.21 mm for *Staphylococcus* spp. (Fig. 3c) and 30.4 ± 0.15 mm for *Streptococcus* spp. (Fig. 3d).

**Table 5:** The antimicrobial efficacy of bacteriocins against biofilm-producing gram-positive cocci

Bacteriocins concentration (µg/mL)	*Staphylococcus* spp. (%)				*Streptococcus* spp*.* (%)										*P* value
	*S. aureus*		*CNS*		*S. agalactiae*		*S. dysgalactiae*		*S. ubris*		*E. faecalis*		*S. lactarius*		
	S	R	S	R	S	R	S	R	S	R	S	R	S	R	
50	50	50	40	60	42.86	57.14	50	50	40	60	50	50	40	60	0.4
100	60	40	60	40	57.14	42.86	50	50	40	60	60	40	50	50	0.2
150	70	30	80	20	71.43	28.57	75	25	60	40	80	20	50	50	0.03
250	90	10	90	10	85.71	14.28	75	25	100	0.0	80	20	75	25	0.05

*S* Susceptible, *R* Resistant; *S. aureus* (*n* = 10)*; CNS* (*n* = 10); *S. agalactiae* (*n* = 7); *S. dysgalactiae* (*n* = 4); *S. ubris* (*n* = 5); *E. faecalis* (*n* = 5); *S. lactarius* (*n* = 2)

All values are expressed in percentages

### Characterization of Biofilm-Forming Bacteria and Anti-Biofilm Activity of Bacteriocins Using FESEM

**Fig.4:**
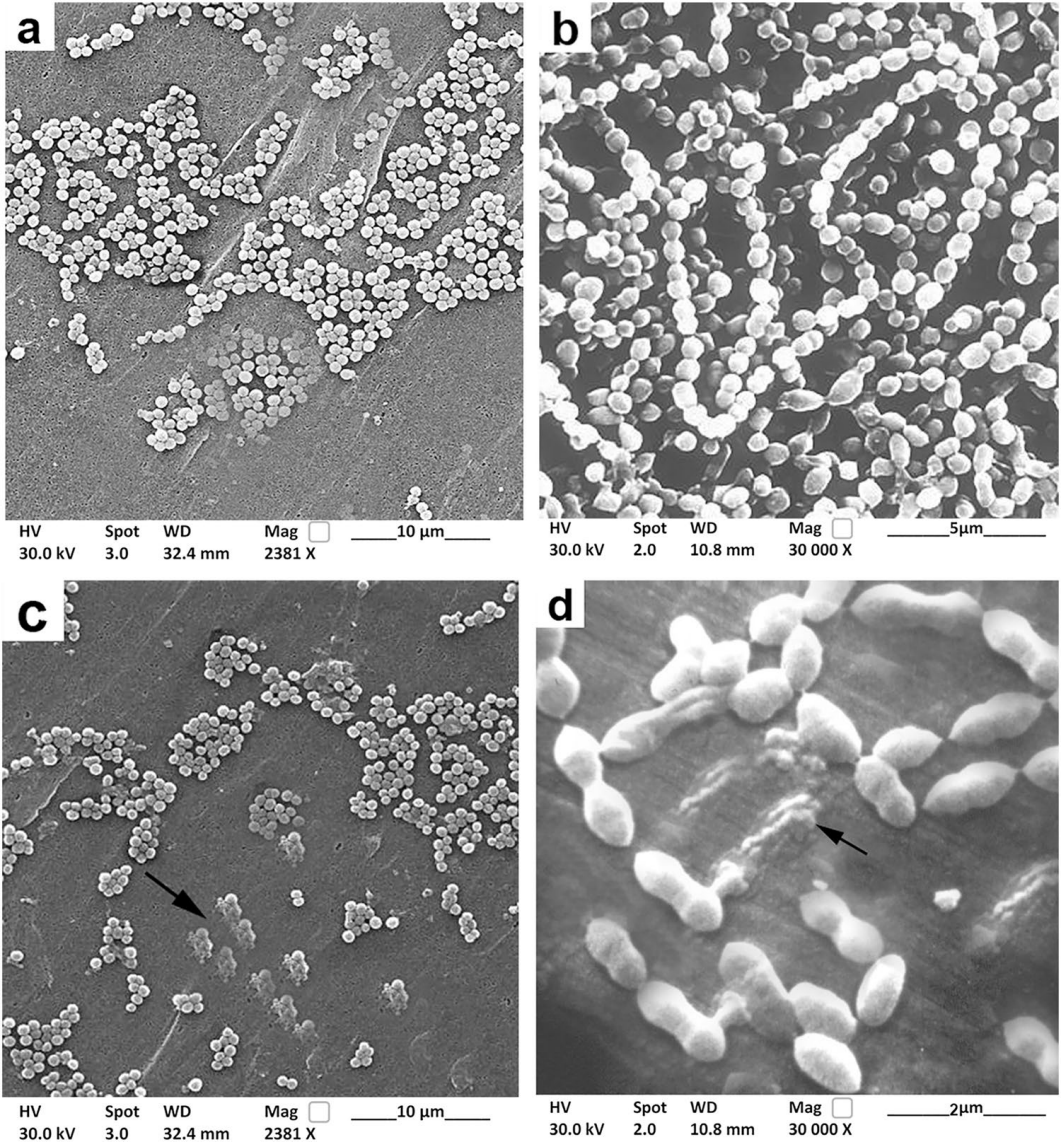
Field emission scanning electron microscopy of biofilm-producing bacteria. The FESEM image of biofilm-producing *Staphylococcus* spp. displayed the normal morphological shape (spherical) as grape-like clusters (**a**). *Streptococcus* spp. appeared as several chains (**b**). The efficacy of bacteriocins against *Staphylococcus* spp. exhibited its action on the bacterial cell (**c**), leading to rupture and damage of the bacterial cell wall. As well, the *Streptococcus* spp. appeared bulged, and the content of bacteria was destroyed (**d**)

FESEM was used to characterize the biofilm-producing pathogenic strains and clarify the anti-biofilm activity of bacteriocins on tested biofilm-forming bacteria (Fig. 4). The FESEM image of biofilm-producing *Staphylococcus* spp. showed the spherical (cocci) form in grape-like clusters (Fig. 4a), while *Streptococcus* spp. appeared in pairs or chains (Fig. 4b). The bacteriocins exhibited their action on *Staphylococcus* spp. (Fig. 4c), leading to rupture and damage of the bacterial cell membrane. Oppositely, the *Streptococcus* spp. bulged, and the content of bacteria was destroyed (Fig. 4d).

## Discussion

Effective programs to manage mastitis are focus more on prevention than on therapy. Antibiotic therapy is still an established part of mastitis prevention regimens today. Even though antibiotics are frequently used in conjunction with other treatments, their effectiveness is still unsatisfactory. Hence, finding novel therapeutics is necessary. Numerous natural remedies derived from plants, animals, and microorganisms have been found capable of controlling bovine mastitis [3].

The prevalence rate of clinical mastitis-causing gram-positive cocci on dairy farms clarified that *Staphylococcus* spp. were the most prevalent isolates in milk of buffalo with mastitis than in milk of cow with mastitis. Contrarily, isolates of *Streptococcus* spp. were more common in the milk of mastitic cows compared to that of mastitic buffalo, as revealed in Table 1. These findings are consistent with Teklemariam et al. [29], who discovered that this variation may be related to the variations in herd management techniques. Some procedures, such as pre- and post-milking teat dipping and pre- and post-milking hand cleaning, have been shown to reduce the incidence of intra-mammary infections. Furthermore, *Staphylococci* and *streptococci* isolates were also examined for enterotoxin-related genes using RT-PCR. The *sed* and *seb* genes were amplified at 278 bp and 194 bp, respectively, while the resistance genes (*mecA* and *blaZ* genes) were amplified at 310 bp and 173 bp, respectively. Previously, El-nomrousey [30] discovered that *sed* and *seb* genes were the most common in all *S. aureus* isolates, while the *mecA* gene was found in 28.5% of them. Awad et al. [31] found the *blaZ* and *mecA* genes in 95.7% and 50% of the *S. aureus* isolates, respectively. Raheel et al. [32] used reverse transcription-PCR to demonstrate that the *sed* and *seb* genes were found in 20% and 80% of *Streptococcus* isolates, respectively, while *mecA* and *blaZ* were found in 90% and 70%, respectively.

The distribution of *Staphylococcus* spp. and *Streptococcus* spp. isolated among the mastitis animals is showed in the current text. The most prevalent *Staphylococcus* spp. isolates discovered in the tested mastitis animals were *CNS* and *S. aureus*, respectively. On the other hand, *S. agalactiae*, *S. dysgalactiae*, and *E. faecalis* were distributed differentially in mastitic cow milk. *S. dysgalactiae* and *E. faecalis*, followed by *S. agalactiae, S. ubris*, and *S. lactarius*, were recorded at the highest concentration in mastitic buffalo milk as displayed in Table 2. El-jakee et al. [33] reported CNS to be the most common pathogens isolated from both clinical and subclinical bovine mastitis in many different countries. According to Wente and Krömker [34],* S. dysgalactiae* is an intermediate pathogen since it may endure both inside as well as outside the host. Zhang et al. [35] isolated *S. dysgalactiae* from 7.5% of their total tested milk samples. Cheng and Han [3] pointed out that contagious bacteria like *S. aureus* can spread quickly and broadly. Environmental infections, on the other hand, can persist without the host and are a natural component of the area around the cow’s microbiota.

The capability of *S. aureus* to produce biofilm had a much higher capacity than that of *CNS* isolates among all biofilm-producing *Staphylococcus* spp. and *Streptococcus* spp. isolates. As well, *S. ubris*, followed by *E. faecalis* and *S. agalactiae*, was the most potent Streptococcus species that produced 100% of the biofilms as shown in Table 3. Additionally, isolated bacteria were shown to contain the genes (*ica A* and *fnb A*) associated with biofilms. Contrarily, *Streptococcus* species that produce biofilms are said to have significant virulence factors. Interestingly, biofilm-producing *Streptococcus* species are said to harbor significant virulence factors [30, 36, 37] in addition to the biofilm-associated genes (*ica*A and *fnb*A), reported in 90% and 70% of the isolates, respectively.

Both Gram-positive and Gram-negative bacteria can release bacteriocins. Those released by lactic acid bacteria are of special relevance to Gram-positive bacteria [38]. A diverse range of facultative anaerobes, acid-tolerant, and fermentative organisms, including lactic acid bacteria, have a ‘qualified presumption of safety’ status, which means the U.S. Food and Drug Administration considers these bacteriocins to be safe [39, 40]. All bacteriocins are amphiphilic and extremely hydrophobic, and they can be purified using a variety of techniques. The standard process includes high-performance liquid chromatography (HPLC-MS), ion exchange chromatography, hydrophobic chromatography on octyl sepharose, and precipitation of the bacteriocins from the culture phase using ammonium sulfate. For instance, low-molecular-weight proteins cannot be precipitated using the widely used ammonium sulfate technique. Even at 75% to 80% saturation, these proteins do not precipitate well, and as they pass through the dialysis sacs, they are completely or partially removed. Because of this, this approach cannot be used to purify low-molecular-weight bacteriocins [41, 42].

Bacteriocins’ antibacterial efficacy against various *Staphylococcus* species was clarified. The sensitivity profile of *S. aureus* to bacteriocins was notably high (100%) and *CNS* (90%) at a concentration of 250 g/mL. Meanwhile, at a concentration of 150 g/mL, its sensitivity to bacteriocins did not exceed 80%. Oppositely, the sensitivity pattern of *Streptococcus* spp. to bacteriocins showed that bacteriocins had a 100% fatal effect on *S. ubris* at 250 g/mL, followed by *S. agalactiae, S. dysgalactiae*, and *E. faecalis*, in that order. *S. lactarius*’ sensitivity to bacteriocins was also lower at the same concentration as clarified in Table 4. According to Perez et al. [43], bacteriocin targets may have a broad spectrum of activity just like antibiotics, blocking multiple biologically significant cell stages. The formation of pores in the cytoplasmic membrane, associated with the mechanism of bacteriocins, is likely caused by dissipation of the proton-motive force, changes in the membrane potential, and changes in the gradient of the proton-positive ion (H^+^). Bacteriocins can also be used *in vitro* to kill or suppress harmful, multidrug-resistant germs [44, 45]. Although bacteriocins have gained popularity as antibacterial peptides against food-borne pathogens [46], they may work better and more broadly when combined with conventional antibiotics [47].

The sensitivity profile of biofilm-producing *Staphylococcus* spp. to bacteriocins at various tested concentrations is showed that biofilm-producing *S. aureus* and *CNS* were highly sensitive to bacteriocins at 250-µg/mL concentration compared to other tested concentrations. Oppositely, the sensitivity profile of *Streptococcus* spp. that produce biofilms revealed that bacteriocins had the most lethal effect against biofilm-producing *S. ubris*, followed by *S. agalactiae*, *E. faecalis*, and *S. dysgalactiae* at 250-µg/mL concentration. In addition, the sensitivity of *S. lactarius*, which produces biofilm, to bacteriocins was not exceeded by 75% at the same concentration. Moreover, the diameter of the inhibitory zone at the greatest dilution (250 µg/mL) was 27.6 ± 0.21 mm for *Staphylococcus* spp. and 30.4 ± 0.15 mm for Streptococcus spp. as shown in Table 5. The skin of the teats acts as a habitat for a rich and diverse microbial community [48]. Bédard et al. [49] and Bennett et al. [50] have showed that bactofencin A has *in vitro* antibacterial activity against *Listeria monocytogenes* and *S. aureus*. Therefore, bactofencin A would be expected to decrease the *Staphylococcus* count more than the *Streptococcus* count and the total viable count. However, in an *in vivo* study, Bennett et al. [7] found that it did not significantly affect any of these counts in comparison to saline. This could be explained by the fact that bacteriocins are susceptible to degradation by proteolytic enzymes [51]. Consequently, the peptide may be vulnerable to the proteases found in teat skin, leading to its degradation.

## Conclusion

This is the first *in vitro* study examining the efficacy of bacteriocins produced by lactic acid bacteria (*B. subtilis*) against biofilm-producing *Staphylococcus* and *Streptococcus* species. Higher bacteriocin concentrations (250 µg/mL) are required to inhibit bacterial growth and control the spread of clinical mastitis-causing bacteria in dairy farms. Bacteriocins are promising candidates for preventing new intra-mammary infections. Further *in vivo* studies are required to more holistically determine the efficacy of bacteriocins in suppressing mammary infections

## Supplementary Information

Below is the link to the electronic supplementary material.

Supplementary file1 (DOCX 15 KB) Supplementary Table 1 Oligonucleotide primer sequences of enterotoxins, resistance, and biofilm genes of isolated bacteria

## Data Availability

All data are included in the main manuscript and are freely accessible.

## Abbreviation

*CNS*: *Coagulase-negative* *Staphylococcus*
*E. faecalis*: *Enterococcus* *faecalis*
*fnbA*: Fibronectin-binding protein A gene
FESEM: Field emission scanning electron microscopy
*icaA*: Intercellular adhesion A gene
*S. aureus*: *Staphylococcus* *aureus*
*S. agalactiae*: *Streptococcus* *agalactiae*
*S. dysgalactiae*: *Streptococcus* *dysgalactiae*
*S. lactarius*: *Streptococcus* *lactarius*
*S. ubris*: *Streptococcus* *ubris*
