# Supplementary material for: The Efficacy of Bacteriocins Against Biofilm-Producing Bacteria Causing Bovine Clinical Mastitis in Dairy Farms: A New Strategy
Source: Curr Microbiol. 2023 May 31;80(7):229. doi: 10.1007/s00284-023-03324-x (PMC10232586; doi:10.1007/s00284-023-03324-x)
Supplement: Supplementary file 1 — Supplementary file1 (DOCX 15 KB) Supplementary Table 1 Oligonucleotide primer sequences of enterotoxins, resistance, and biofilm genes of isolated bacteria [file 284_2023_3324_MOESM1_ESM.docx]

**Article title: The Efficacy of Bacteriocins against Biofilm-Producing Bacteria Causing Bovine Clinical Mastitis in Dairy Farms: A New Strategy**

**Journal name: Current Microbiology**

**Author names: Ismail Raheel^1^. Asmaa N. Mohammed^2^. Asmaa Abdrabo Mohamed^3^**

**Affiliation**^: 1^Department of Bacteriology, Mycology and Immunology, Faculty of Veterinary Medicine, Beni-Suef University, Beni-Suef 62511, Egypt.

^2^Department of Hygiene, Zoonoses and Epidemiology, Faculty of Veterinary Medicine, Beni-Suef University, Beni-Suef 62511, Egypt.

^3^Veterinarian at the Directorate of Veterinary Medicine, El-Fayoum Governorate, Egypt.

**Email of corresponding author**: asmaa.mohamed2@vet.bsu.edu.eg

**Supplementary Table 1 Oligonucleotide primer sequences of enterotoxins, resistance and biofilm genes of isolated bacteria.**

| Target gene (Primers) | Primer sequences 5’-3’ | Amplified product | Reference |
| --- | --- | --- | --- |
| Enterotoxins genes  *Sed* | CCAATAATAGGAGAAAATAAAAG | 278 bp | Mehrotra et al. [19] |
|  | ATTGGTATTTTTTTTCGTTC |  |  |
| *Seb* | GTATGGTGGTGTAACTGAGC | 164 bp |  |
|  | CCT AAC TAA CGA AAG GTA G |  |  |
| Resistance genes  *mecA* | GTA GAA ATG ACT GAA CGT CCG ATA A | 310 bp | McClure et al. [20] |
|  | CCA ATT CCA CAT TGT TTC GGT CTA A |  |  |
| *blaZ* | ACTTCAACACCTGCTGCTTTC | 173 bp | Duran et al. [21] |
|  | TGACCACTTTTATCAGCAACC |  |  |
| Biofilm genes  *ica*A | CCT AAC TAA CGA AAG GTA G | 1315 bp | Ciftci *et al.* [23] |
|  | AAG ATA TAG CGA TAA GTG C |  |  |
| *fnb*A | CAT AAA TTG GGA GCA GCA TCA | 127 bp | Vancraeynest *et al.* [24] |
|  | ATC AGC AGC TGA ATT CCC ATT |  |  |
